# Clinical consequences of changing the sliding window IMRT dose rate

**DOI:** 10.1120/jacmp.v13i4.3810

**Published:** 2012-07-05

**Authors:** E. Ghasroddashti, W.L. Smith, S. Quirk, C. Kirkby

**Affiliations:** ^1^ Jack Ady Cancer Center Lethbridge Alberta; ^2^ University of Calgary Department of Physics and Astronomy Calgary Alberta; ^3^ University of Calgary Department of Oncology Calgary Alberta; ^4^ Tom Baker Cancer Centre Calgary Alberta Canada

**Keywords:** IMRT, dose rate, monitor units, radiation safety

## Abstract

Changing pulse repetition frequency or dose rate used for IMRT treatments can alter the number of monitor units (MUs) and the time required to deliver a plan. This work was done to develop a practical picture of the magnitude of these changes. We used Varian's Eclipse Treatment Planning System to calculate the number of MUs and beam‐on times for a total of 40 different treatment plans across an array of common IMRT sites including prostate/pelvis, prostate bed, head and neck, and central nervous system cancers using dose rates of 300, 400 and 600 MU/min. In general, we observed a 4%–7% increase in the number of MUs delivered and a 10–40 second decrease in the beam‐on time for each 100 MU/min of dose rate increase. The increase in the number of MUs resulted in a reduction of the “beam‐on time saved”. The exact magnitude of the changes depended on treatment site and planning target volume. These changes can lead to minor, but not negligible, concerns with respect to radiation protection and treatment planning. Although the number of MUs increased more rapidly for more complex treatment plans, the absolute beam‐on time savings was greater for these plans because of the higher total number of MUs required to deliver them. We estimate that increasing the IMRT dose rate from 300 to 600 MU/min has the potential to add up to two treatment slots per day for each IMRT linear accelerator. These results will be of value to anyone considering general changes to IMRT dose rates within their clinic.

PACS number: 87.55.N

## I. INTRODUCTION

Recently our two cancer centers have made the decision to increase the dose rate (specifically, the pulse repetition frequency) used in conventional (nonVMAT), sliding window IMRT from 300 MU/min to 600 MU/min. This decision was partially motivated by a desire to reduce overall treatment delivery times and increase the number of treatment slots per day. Reducing treatment times also has the advantage of reducing dosimetry errors that may result from intrafraction organ motion and/or volume changes correlated with time.^(^ ^1^ ^–^ ^3^ ^)^ One consequence of increased dose rate was a general increase in the total number of monitor units (MUs) delivered during each fraction. In turn, this led to questions and concerns over the consequences of such an increase. These included: (i) radiation protection concerns, (ii) concerns about increased whole body dose to patients from head leakage, and (iii) concerns about the increased relevance of various approximate parameters used by our shared treatment planning system such as interleaf leakage. To investigate these, we first established a general relationship between number of MUs and dose rate for various treatment sites.

The precise relationship between dose rate, delivery time, and the number of MUs required to deliver a desired dose distribution is a complex one.^(^ ^4^ ^)^ In sliding window leaf motion calculation (LMC) algorithms, the desired fluence pattern is broken into segments (the elements between control points), which are each characterized by a critical “speed” in cm/MU. This critical “speed” is defined by dividing the mechanical leaf speed in cm/s by the dose rate in MU/s. Across a segment, a difference in mechanical speeds between the leading and following leaves is introduced. The total number of MUs for which the source is directly exposed between the leading and trailing leaves defines the relative transmitted fluence for that segment. The maximum mechanical leaf speed (cm/s) then introduces a limitation to the process. When the dose rate (MU/s) is increased, the number of segments defined by the critical speed increases and, as a result, the total number of MUs required to deliver the plan goes up.

Naturally, the number of MUs delivered for a given IMRT plan is going to be dependent to varying degrees on planning system‐specific traits including the algorithm used to translate a desired fluence map into MLC leaf motions (the leaf‐motion calculator or LMC), the physical characteristics of the accelerator head and the MLC, and the characteristics of the model of the MLC within the treatment planning system (TPS) and the source model. The number of MUs will also be dependent on plan‐specific traits such as the characteristics of the desired fluence maps and, therefore, on the treatment sites and the planning approach used. Thus, an exhaustive approach to determining the precise increase in MUs associated with an increase in dose rate is not a practical reality. The problems introduced by an increase in MUs still warrant attention, however.

In this work, we present a survey of the relationship between dose rate and number of MUs across several common IMRT treatment sites, examining ten cases for each. The results are particular to the TPS, LMC, linear accelerators, and planning approach used at our centers, but may serve either as a baseline for general comparison, or provide clinical physicists with an approximate magnitude of the relationship and standard deviation therein for making their own decisions about IMRT dose rates.

## II. MATERIALS AND METHODS

All plans were created using Varian's Eclipse TPS incorporating the Anisotropic Analytical Algorithm (AAA) version 8.9.08 and the associated LMC version 8.9.08 (Varian Medical Systems, Palo Alto, CA).^(^ ^5^ ^)^ The MLC modeled was Varian's Millennium 120 mounted on a Varian iX linear accelerator and using a 6 MV photon source calibrated (per TG‐51 protocol^(^ ^6^ ^)^ to deliver 1.000 cGy/MU to $d_{\max}$ for a $10\times 10\;{\text{cm}}^{2}$ field in a 100 cm SAD setup. Specific properties of the system relevant to the MU calculations are detailed in Table 1.

**Table 1:** Properties of the MLC system used in this work.

*Property*	*Value*
MLC Model	Varian Millennium 120
Maximum leaf span	15.0 cm
Maximum leaf speed	2.5 cm/s
Dosimetric leaf gap	0.15 cm
Interleaf leakage	1.5 %
Leaf transmission	2.5 %
Controller Software Version	7.4.1.6

We considered the following five treatment sites: (i) prostate/whole pelvis, (ii) prostate bed, (iii) head and neck (H&N), (iv) central nervous system (CNS), and (v) gastrointestinal tract. For each site, ten completed clinical plans were randomly selected from the database. The plans were generated and treated at a dose rate of 300 MU/min. For each case, the clinical plan was copied twice. In the second copy, the dose rate was increased to 400 MU/min and in the third, it was increased to 600 MU/min. The fluence patterns determined during the initial optimization process were not modified. For each of the two new versions of the plan, the LMC was rerun for each field, and the final dose distribution was recalculated. Towards the end of the planning process, the TPS splits fields that are greater than 13.5 cm along the direction of MLC motion into subfields to account for the maximum allowable leaf travel across the field.

To effectively compare the difference in delivered MUs, we tallied the total number of MUs required deliver a complete treatment fraction for all fields (and/or subfields), and normalized that to the prescribed dose for a given fraction. Our raw results were given in units of MU/cGy. To more clearly depict the changes, we further normalized the raw results to the 300 MU/min dose rate. Site‐specific details of the prescriptions are given below.

Treatment delivery times were also calculated to estimate, in practical terms, the amount of time saved by increasing the dose rate. These were calculated as the total number of monitor units divided by the dose rate multiplied by a factor of 1.05. The 5% increase factor is applied to account for delays due to beam stops or fluctuations in instantaneous dose rate over the course of treatment. This factor was based on direct stopwatch measurements of a subset of plans, including each treatment site investigated, which had a mean value of 1.051. We note that this factor is separate from the planning time factor applied to treatment plans to set the time interlock, which is normally not set as a mean value, but as an extremely high value so as not to commonly trip before the natural complete delivery of a field, but not so high as to place the patient at risk from extended delivery in the event the MU dosimetry interlocks fail. Based on an internal survey of treatments at our centers, IMRT treatment timelines can be broken down approximately as follows: 5 minutes for patient setup and administration, 3–4 minutes for image‐guided setup (cone‐beam CT‐based 3D‐3D image matching), 4–8 minutes of actual treatment time including gantry and collimator transitions, split field transitions, and beam‐on time (1.5–3 minutes depending on dose rate), and 3 minutes for takedown. The treatment delivery times discussed herein were considered beam‐on times only. They did not account for patient setup, positioning, nursing, record and verify quality control, or transit time between fields, and thus account for approximately 20% of total treatment times.

The details of the prescriptions and the planning process for each site are naturally going to determine the absolute numbers of monitor units arrived at for each specific plan. Here, we provide a brief overview of the planning process for the plans involved.

The prostate/whole pelvis plans were treated in two phases: phase one — where the pelvic lymph nodes were included in the planning target volume, and phase two — where the fields were coned down to a second PTV expanded from the prostate and seminal vesicles. Each phase consisted of either 5 or 7 treatment fields (broken into subfields when demanded by treatment area) with collimator angles optimized for efficiency. PTV volumes ranged from ~400 to 1400 cc (phase one) and from ~120 to 400 cc (phase two). Prescriptions per fraction ranged from 180 to 200 cGy (phase one) and were consistently 200 cGy (phase two), with a goal of covering 95% of the PTV with these isodose lines, while subjecting planning risk volumes including rectum, bladder, body, and femoral heads to local dose‐volume constraints generally consistent with RTOG guidelines.

The ten prostate bed plans were treated in a single phase consisting of either 5 or 7 fields. The planning approach was similar to that for the prostate/whole pelvis case. PTV volumes ranged from ~250 to 500 cc, and the prescribed dose per fraction ranged from 180 to 262.5 cGy, again with a goal of covering 95% of the PTV with these isodose lines, while meeting local dose‐volume constraints.

Cancers of the head and neck were also examined, including cancers of the nasopharynx, cheek, tonsils, tongue, and neck. As expected, these plans were considerably more complex than the prostate or prostate bed cases, consisting often of 2 PTVs (e.g., a subvolume prescribed to 6000 cGy within a larger volume prescribed to 5400 cGy where the planning goal was to deliver the prescribed dose to at least 95% of the identified volume). PRVs were also more abundant than the prostate cases, including such structures as eyes, optic nerves, optical chiasm, brain stem, spinal cord, parotid glands, cochlea, mandible, larynx, and pharynx. The number of fields ranged from 6–7 depending on the site, with collimator rotation optimized. Where the treatment consisted of multiple phases, only the first phase was investigated. Dose per fraction ranged from 212.1 to 220 cGy.

The CNS cases targeted various PTVs within the brain. In general, they presented somewhat less complexity with respect to the overall number of critical structures compared to the H&N cases, but PRVs still included such structures as the eyes, optical chiasm, optic nerves, brainstem, spinal cord, parotid glands, and cochlea. The number of fields ranged from 5–6 depending on the site, with collimator rotation optimized. The range for prescribed doses per fraction ranged from 180 to 266.7 cGy.

To ensure dosimetric consistency between plans where the dose rate had been changed, we measured one plan per site, all dose rates, using our local plan‐specific IMRT validation protocol across two separate treatment units. Under this protocol, each field in the plan was projected onto a verification water block phantom in the treatment planning system. Dose maps in plane with isocenter (with 7 cm of buildup) were exported. Comparison measurements were performed using the MapCHECK2 diode array (Sun Nuclear Corporation, Melbourne, FL) and associated software. The MapCHECK2 was placed in plane with isocenter, with the gantry at 0° and collimator at 0° for all fields. Five cm of solid water plus 2 cm of water‐equivalent intrinsic material, made up the 7 cm buildup. Plans were delivered by Varian iX linear accelerators with Millennium 120 leaf MLCs running MLC Controller software version 7.4.1.6. The measured dose maps were evaluated against those predicted by the treatment planning system using a 3%/3 mm acceptance criteria down to a threshold of 10 cGy. Comparisons were made using absolute dose with no correction for daily fluctuation in output, which ranged from 0.996 to 1.015 cGy/MU (to water at $d_{\max}$ for $10\times 10\;{\text{cm}}^{2}$ fields, SAD geometry) on the machines used. Prior to these measurements, we confirmed the independence of the MapCHECK2 diodes with respect to dose rate by measuring 100 MU $10\times 10\;{\text{cm}}^{2}$ open fields in the same setup with an A12 ion chamber and electrometer at 1.5 cm depth in the solid water. From 300 to 600 MU/min differences of the central axis diode with the relative ion chamber readings were less than 0.5%.

## III. RESULTS

**Figure 1:**
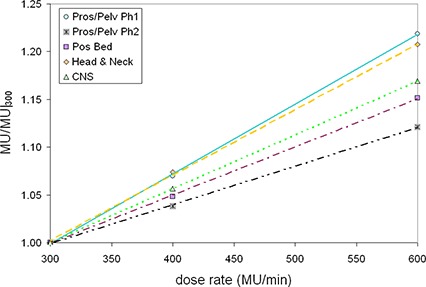
The mean trends in relative number of MU (normalized to the 300 MU/min doe rate) as a function of dose rate for all sites investigated. The maximum standard deviation in the 600 MU/min rate was 0.04 for both the phase one prostate/ pelvis and the brain cases.

**Figure 2:**
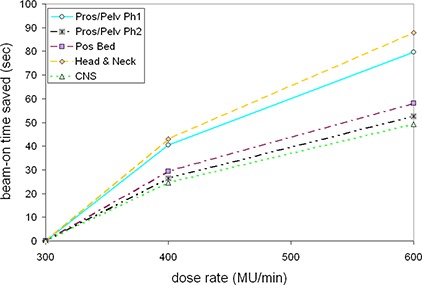
The mean trends in beam‐on time saved (as a difference from the 300 MU/min doe rate) as a function of dose rate for all sites investigated. The trend is not linear because, as the dose rate increases, more MUs are required to deliver the same fluence pattern.

Mean results for each site investigated are presented in Table 2. The trends in relative monitor unit increase and “beam delivery time saved” are then presented in Figs. 1 and 2, respectively.

**Table 2:** A summary of the results across all sites for dose rates (in MU/min). Presented are the mean and standard deviation across the ten plans considered.

*Site*		*300*	*MU D/fx 400*	*600*	*300*	*Delivery Time (min) 400*	*600*
Prostate Pelvis Ph1	mean	5.38	5.76	6.58	3.41	2.74	2.08
	std	1.01	1.09	1.31	0.58	0.47	0.38
Prostate Pelvis Ph2	mean	2.85	2.95	3.19	1.99	1.55	1.12
	std	0.49	0.50	0.54	0.34	0.26	0.19
Prostate Bed	mean	3.26	3.41	3.75	2.28	1.79	1.31
	std	0.84	0.89	1.00	0.49	0.38	0.28
Head & Neck	mean	4.92	5.29	5.94	3.70	2.98	2.23
	std	0.75	0.82	0.91	0.56	0.46	0.34
Central Nervous	mean	2.86	3.03	3.36	1.98	1.57	1.16
	std	0.59	0.67	0.78	0.33	0.28	0.22

In Fig. 1 we plot the mean increase in MUs relative to the 300 MU/min dose rate. As expected, for each of the sites investigated, there was a clear linear increase in the number of MUs with each increased dose rate. MU increases appeared to have a dependence on the specific site or phase of treatment. Expressed in terms of percentage increase in MUs per 100 MU/min increase in dose rate, we observed gains of 7.0%±1.0% for prostate/pelvis phase one, 4.1%±0.6% for prostate/pelvis phase two, 5.0%±1.0% for prostate bed, 6.9%±0.8% for the H&N cases, and finally 6.0%±1.0% for the CNS cases. Qualitatively speaking, it is reasonable to conclude that as the complexity of the plans increased, so did the relative number of MUs. Further, for gross workload estimates, it seemed reasonable to anticipate an increase in MU workload of approximately 20% across all IMRT sites when increasing the dose rate from 300 to 600 MU/min.

The associated beam‐on time saved was expressed as a difference from the 300 MU/min case in seconds; the mean trends are presented in Fig. 2. It is obvious that the time saved is not linear with dose rate because the number of MUs required to deliver the same final dose distribution increases with dose rate. Thus, doubling the dose rate from 300 to 600 MU/min did not half the beam delivery time. Rather, it was reduced to approximately: 60%±2% for prostate/pelvis phase one, 56%±1% for prostate/pelvis phase two, 58%±2% for prostate bed, 60%±1% for H&N, and 59%±2% for the CNS cases. On a practical level, this translated into time savings of between 50–90 seconds per treatment in moving from 300 to 600 MU/min.

**Figure 3:**
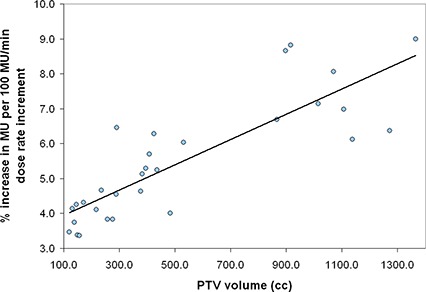
The percentage increase in MU per 100MU/min increase in dose rate as a function of PTV volume in cc for the prostate cases. The black line is the linear regression fit to the data. It increases at a rate of ~ 0.36% per 100 cc of volume increase.

The prostate cases (phase one and two of the prostate pelvis and the prostate bed treatments) all followed reasonably similar approaches to planning with similar risk volumes for avoidance and, thus, suggested a possible correlation between monitor unit increase and target volume. In Fig. 3, we show that the increase in MU with increasing dose rate correlates with PTV size. This figure shows the percentage increase in MU per 100MU/min increase in dose rate against PTV volume in cc. Over the volumes investigated, we observed relative increases in MU between 4%–9% for each 100 MU/min dose rate increase. A regression fit to the data suggests an approximate increase in MU of 0.36% per additional 100 cc of planning volume considered. This is not surprising, as larger volumes require larger area fluences and thus more control points.

**Figure 4:**
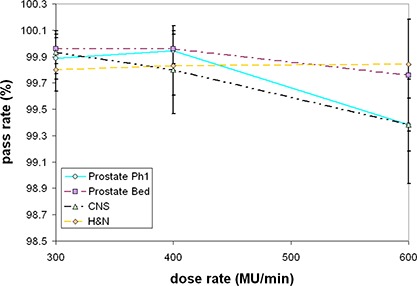
The measured pass rates (mean across all fields, error bars indicate one standard deviation) for a subset of plans meeting a 3%/ 3 mm acceptance criteria. A 95% pass rate is considered clinically acceptable. This demonstrates the plans are delivering dose maps clinically consistent with the treatment planning system prediction generated using a dose rate of 300MU/min.

In Fig. 4 we present a graph summarizing the results of the plans measured using our IMRT validation protocol. The values shown are the mean passing points expressed as a percentage of planned doses over 10 cGy, across all fields of the respective plan. The error bars indicate plus or minus one standard deviation. Generally, we use a 5% failure rate (for each field) to identify a clinically unacceptable plan. The worst case was 98.7% of the points passing for one of the brain fields measured at 600 MU/min. All cases at all dose rates measured would have passed our criteria for a clinically acceptable plan. While changing the dose rate forces the LMC to be rerun and generates a different MLC control file, based on these measurements, we established that changing the dose rate does not appear to introduce changes in the final fluence pattern that result in clinically relevant differences in dose distribution.

## IV. DISCUSSION

In this work, we established that using the different dose rates, measured 2D dose maps are similar within clinically acceptable criteria. Examining Fig. 4, for three of the cases there may be a slight downward trend in the data as dose rate is increased. This is to be expected, as in all cases we were comparing the final measured dose map at a given dose rate to the calculated dose map generated using the fluence pattern from the 300 MU/min plan. Clinically, it is advisable and only fair to compare the measurement to the plan for the dose rate at which it was delivered. We would expect a 600 MU/min measurement to conform more closely to a 600 MU/min plan. The approach used here is not meant to be an exhaustive examination of subtle differences in delivered fluence patterns at different dose rates. Indeed, there is evidence to suggest increasing dosimetric uncertainty at higher dose rates for certain IMRT planning and delivery control systems.^(^ ^7^ ^,^ ^8^ ^)^

Our results indicate that systematically increasing the dose rate for conventional IMRT treatments leads to a corresponding MU increase of approximately 4%–7% per 100MU/min increase. The exact factor has dependencies on treatment site and the irradiated volume. In jumping from 300 to 600 MU/min, it is not unreasonable to expect the number of MUs within a facility to increase by over 20%, which can potentially lead to several clinical concerns.

With respect to the time savings, 50–90 seconds per patient may not at first seem significant. However, for a treatment unit that treats 30 patients per day, this translates into anywhere from 25 to 45 minutes of available machine time and can open up 1–2 more treatment slots. From a population point of view, this can cascade into reduced wait‐times, which potentially improves treatment outcomes.^(^ ^9^ ^)^ However, a certain flexibility in booking is required to take advantage of this time savings, and care must be taken that the time saved in delivery is used to increase patient throughput.

The observed increase in MU workload can potentially be cause for a re‐evaluation of bunker shielding from a radiation protection point of view, depending on how liberal workload estimates were at the time the facility in question was designed and constructed (or redesigned and modified in the case of vaults that have seen multiple generations of linacs). In addition to the direct gain in MUs, a secondary effect comes into play, as well. As discussed above, treatment time savings can cause centers to increase the number of patients treated on a unit. Thus workload estimates increase in two places: the IMRT factor and the number of patients per day. In the case of moving from 300 MU/min to 600 MU/min, it would be prudent to increase the IMRT factor by 20% and the number of patients increased by roughly 2/25 or 8%; meaning the overall workload would increase by about 30%.

A medical question that came to light during these discussions in our center had to do with induction of secondary cancers. Given that the demographic of radiotherapy patients tends to be older than the general population, Hall and Wuu^(^ ^10^ ^)^ suggest a value of 2%/Gy as a risk estimate for the induction of secondary cancers resulting from radiotherapy. If we assume that the dose to a patient from head leakage radiation delivers ~0.001 cGy/MU,^(^ ^11^ ^)^ the risk of inducing secondary cancers from head leakage becomes ~0.00002%/MU. Thus, a prostate patient whose total number of MUs has increased by 20% (from 40,000 to 48,000) as a consequence of the clinic adopting a new dose rate, will have the probability of head leakage inducing a secondary cancer move from ~0.8% to 0.96%. This does not push the probability of inducing a secondary cancer into an unacceptable range; however, it may not necessarily be insignificant.

From a planning point of view, it is important to remember that MLC leaf transmission can be approximately 1%–2%, and interleaf leakage can allow through up to 4% of the intensity of the radiation incident on the MLC. Since, in a sliding window IMRT plan regions of low fluence are delivered using the MLC leaves as shields, increasing the number of MUs in a plan degrades the minimum fluence permissible by a fluence pattern. This leads to an increased dependence on the accuracy of the source model used in the treatment planning system. Further, even if the source model were perfect, more radiation will leak through the leaves, which has the potential for introducing minor planning restrictions (i.e., a less optimal dose distribution with increased doses to PRVs or even increased probability of secondary cancer induction may result from an increased dose rate).

## V. CONCLUSIONS

Increasing pulse repetition frequency or dose rate for conventional sliding window IMRT treatment delivery has a number of practical consequences. A survey across an array of common IMRT sites including prostate/pelvis, prostate bed, H&N, and CNS cancers, showed increases in the number of MUs delivered of approximately 4%–7% per 100 MU/min of dose rate increase. We observed dependencies of these changes on treatment site and PTV size. This increase subsequently reduces the “beam‐on time saved” that had resulted from increasing the dose rate; however, the net result is still a shortened overall treatment time. Therefore, large changes in dose rate can potentially open enough time to increase the number of patients treated in a day.

In our centers, it was decided that the increased patient throughput and decreased intrafraction motion outweighed the clinical consequences of the increase in MUs. However, we recommend that any center considering such a change in dose rate should conduct its own independent review of the increase in workload in light of the patient population treated at that center.
